# Prognostic utility of the global leadership initiative on malnutrition criteria in predicting outcomes after hepatectomy for hepatocellular carcinoma: a multicenter, retrospective study

**DOI:** 10.3389/fnut.2025.1610066

**Published:** 2025-06-26

**Authors:** Yaohao Liang, Zhan Lu, Tianyu Ruan, Shanglin Wu, Jianyi Meng, Jie Jiang, Jiaqian He, Liyan Jiang, Ning Tan, Shengqiang Tan

**Affiliations:** ^1^Department of Hepatobiliary and Pancreatic Surgery, Nanning Ninth People’s Hospital, Nanning, China; ^2^Department of Hepatobiliary and Pancreatic Surgery, Liuzhou People’s Hospital Affiliated to Guangxi Medical University, Liuzhou, China; ^3^Department of Hepatobiliary and Pancreatic Surgery, Guangxi Medical University Cancer Hospital, Nanning, China; ^4^Department of Radiology, Liuzhou People’s Hospital Affiliated to Guangxi Medical University, Liuzhou, China; ^5^Guangxi Key Laboratory of Diabetic Systems Medicine, College of Basic Medical Sciences, Guilin Medical University, Guilin, China; ^6^Clinical Nutrition Department, Liuzhou People’s Hospital Affiliated to Guangxi Medical University, Liuzhou, China

**Keywords:** hepatocellular carcinoma, global leadership initiative on malnutrition, hepatectomy, prognosis, predictive model

## Abstract

**Objective:**

The Global Leadership Initiative on Malnutrition (GLIM) criteria were developed to standardize diagnoses of malnutrition. However, the prognostic utility of the GLIM criteria and predictive models including GLIM criteria in patients diagnosed with hepatocellular carcinoma (HCC) undergoing hepatectomy remains largely unexplored.

**Methods:**

This retrospective study included 477 HCC patients who underwent curative hepatectomy at two centers (training cohort: *n* = 297, January 2014 to November 2020; validation cohort: *n* = 180, April 2018 to December 2019). A nomogram was developed using multivariate Cox regression analysis. The utility of the developed model was evaluated by Harrell concordance index (C-index), calibration curve, and decision curve analysis (DCA). Time-dependent receiver operating characteristic (ROC) curves and DCA were used to compare the nomogram with existing prognostic models.

**Results:**

The prevalence of malnutrition was 30.6 and 25.6% in the training and validation cohorts, respectively. Non-malnourished patients exhibited superior overall survival (OS) across all BCLC stages (*p* < 0.001). Multivariate analysis identified GLIM-diagnosed malnutrition, albumin <35 g/L, tumor size >5 cm, alpha-fetoprotein (AFP) ≥400 ng/mL, and tumor number ≥3 as independent predictors of OS. The nomogram for 3-year OS achieved C-indices of 0.735 and 0.666 in the training and validation cohorts, respectively. External validation demonstrated good discrimination and calibration. The nomogram outperformed the ALBI, PNI, and BCLC staging systems in terms of AUC and DCA.

**Conclusion:**

GLIM-diagnosed malnutrition was an independent risk factor for OS in patients with HCC undergoing hepatectomy. The nomogram including GLIM is a good tool for predicting postoperative OS in this patient population.

## 1 Introduction

Hepatocellular carcinoma (HCC) is the most common primary liver malignancy worldwide, accounting for 75–85% of all primary liver cancer cases (1). Treatment options for HCC include liver resection, local ablation, transarterial chemoembolization (TACE), targeted therapy, and immunotherapy. Among these, liver resection is the most widely used curative treatment. However, the five-year overall survival rate after surgery is only 51% (2, 3), indicating an unsatisfactory prognosis. Numerous factors influence the prognosis of HCC patients undergoing resection, including tumor characteristics, liver function, and nutritional status. Among these, malnutrition is closely associated with increased postoperative complications, prolonged hospital stays, higher medical costs, and reduced long-term survival rates (4, 5). Studies show that malnutrition is a major cause of 10–20% of cancer-related deaths (4, 5), highlighting its significance in cancer treatment. Importantly, nutritional status is one of the few modifiable factors that can be addressed before surgery. Given the significant impact of malnutrition on cancer prognosis, accurate nutritional assessment is crucial for optimizing treatment outcomes.

Various tools, such as the Patient-Generated Subjective Global Assessment (PG-SGA), the Nutritional Risk Screening 2002 (NRS-2002), and the Malnutrition Universal Screening Tool (MUST), have been developed to assess nutritional status (6, 7). However, these tools have limitations, including complex assessment processes, neglecting key nutritional factors (e.g., skeletal muscle mass), and lack of standardization across regions (8). To overcome these issues, the Global Leadership Initiative on Malnutrition (GLIM) criteria were introduced in 2018 by several clinical nutrition societies worldwide (9). The GLIM criteria integrate phenotypic criteria, which reflect physical signs of malnutrition (e.g., weight loss, low BMI, reduced muscle mass), and etiologic criteria, which address underlying causes (e.g., reduced food intake, inflammation, disease burden). Malnutrition is diagnosed when at least one of each type is present. Compared to PG-SGA, GLIM is more concise and easier to use, and its diagnostic consistency has been validated in relevant studies (10, 11).

Although the application of GLIM criteria is increasing in oncology surgery (12–15), their utility in HCC patients undergoing resection remains under-explored, particularly regarding predictive models for postoperative survival outcomes. Therefore, this study aims to explore the prognostic value of GLIM criteria in HCC patients undergoing resection and to develop a novel predictive nomogram incorporating these criteria to forecast postoperative survival outcomes. The findings of this study are expected to provide clinicians with a basis for preoperative nutritional assessment and intervention, thereby optimizing treatment plans and postoperative quality of life for HCC patients undergoing resection.

## 2 Materials and methods

### 2.1 Study design

This multi-center retrospective study analyzed the data from patients with HCC who underwent hepatectomy at two institutions. The training cohort included eligible patients treated at Liuzhou People’s Hospital affiliated with Guangxi Medical University (Guangxi, China) between January 2014 and November 2020. This cohort was used to develop a predictive model for 3-year OS. The validation cohort used to assess the applicability of the model comprised eligible patients treated at the Affiliated Tumor Hospital of Guangxi Medical University between April 2018 and December 2019. Subgroup analyses were performed across Barcelona Clinic Liver Cancer (BCLC) stages to evaluate the impact of nutritional status on OS across disease stages.

The sample size for the training cohort was designed in accordance with the recommended standards for developing Cox regression models (16), setting the events per variable (EPV) ratio to 10–15 to mitigate the risk of overfitting and enhance the robustness and reliability of parameter estimates. We determined the required number of events based on the number of candidate predictors. The sample size for the validation cohort was designed following the guidelines of Collins et al. (17) to ensure statistical reliability in performance evaluation, requiring a minimum of 100 events to achieve unbiased estimation and high precision of discrimination metrics (such as the C-index), while also assessing the comparability of data across institutions.

### 2.2 Study population

The inclusion criteria were as follows: (1) clinically and pathologically confirmed HCC; (2) complete preoperative nutritional and clinical data; (3) and comprehensive medical records and follow-up information. Exclusion criteria included: (1) extrahepatic metastasis, preoperative infection or inflammatory diseases; (2) concomitant malignancies; (3) systemic diseases (e.g., hematological or autoimmune disorders); (4) death within 30 days postoperatively; (5) failure to fulfill curative resection criteria; (6) emergency surgery due to ruptured HCC; (7) incomplete nutritional assessment data or clinical data; (8) preoperative trans-arterial chemoembolization (TACE) or postoperative targeted/immunotherapy. This study adhered to the Declaration of Helsinki and was approved by the Ethics Committee of Liuzhou People’s Hospital affiliated with Guangxi Medical University (approval number: KY2022-030-02).

### 2.3 Data collection

The following patient information was extracted from electronic medical records: (1) demographic data (sex, age, height, weight); (2) nutritional assessment [body mass index (BMI), unintentional weight loss, computed tomography (CT)-based L3 skeletal muscle index (L3-SMI), GLIM criteria]; (3) tumor characteristics [maximum diameter, number, microvascular invasion (MVI)]; (4) surgical details (extent of hepatectomy and length of hospital stay); (5) preoperative blood tests within 1 week before surgery.

### 2.4 Definitions

The criteria for curative hepatectomy were as follows: (1) complete resection of all tumor nodules identified preoperatively and intraoperatively with negative microscopic margins; (2) no preoperative evidence of extrahepatic metastasis; (3) for patients positive for alpha-fetoprotein (AFP)-positive, normalization of AFP within 2 months postoperatively (excluding elevation due to hepatitis or cirrhosis); (4) no characteristic tumor findings on imaging studies.

Nutritional assessment was performed using the GLIM criteria, which require meeting at least one phenotypic criterion and one etiologic criterion, as detailed in Table 1. In this study, “malnutrition” specifically refers to malnutrition diagnosed according to the GLIM criteria.

**Table 1:** GLIM criteria and diagnostic procedures.

Step	Content	
Step 1: nutritional risk screening	Content: utilize a clinically validated screening tool to identify individuals at risk of malnutrition	
Step 2: assessment of malnutrition (diagnosis: malnutrition is diagnosed when at least one phenotypic criterion and one etiologic criterion are met)	Phenotypic criteria	
	Unintentional weight loss	5–10% weight loss within 6 months, or 10% weight loss beyond 6 months.
	Low body mass index (BMI)	Asia: <18.5 kg/m^2^ (age <70 years), <20 kg/m^2^ (age ≥70 years)
	Reduced muscle mass	Below normal values as determined by various body composition measurement methods
	Etiologic criteria	
	Reduced food intake or absorption	Food intake reduced by <50% for more than 1 week, or reduced intake persisting for >2 weeks, or chronic gastrointestinal dysfunction impairing food digestion and absorption
	Disease burden or inflammatory state	Associated with acute disease or trauma, or associated with chronic disease
Step 3: severity grading	Assess the severity of malnutrition based on the phenotypic criteria	

L3-SMI assessment involved abdominal CT performed within 72 h of admission. The total skeletal muscle area at level of L3 was measured, including the psoas, erector spinae, quadratus lumborum, transversus abdominis, external and internal oblique, and rectus abdominis muscles. The Japanese Society of Hepatology’s guidelines for sarcopenia in liver disease (1st edition) recommend using CT to measure the L3-SMI, with the criteria for low muscle mass being <42 cm^2^/m^2^ in men and <38 cm^2^/m^2^ in women (18). This study adopts these criteria as the reference standard for assessing reduced muscle mass.

### 2.5 Postoperative follow-up

The initial follow-up occurred 4–6 weeks postoperatively, with 1–2 adjuvant TACE sessions for high-risk patients. Subsequent follow-ups were performed every 3–6 months for the first 2 years, then every 6–12 months thereafter, including medical history, physical examination, and AFP testing. Abdominal CT or liver magnetic resonance imaging was performed every 6 months for the first 2 years, and annually thereafter. Recurrence or metastasis was diagnosed based on imaging and clinical data, with confirmation by biopsy when necessary. Follow-up data were obtained from the disease-management offices of both participating hospitals. OS was defined as the interval from diagnosis to death from any cause or the last follow-up. The final follow-up date was August 31, 2022.

### 2.6 Handling of missing data

To address potential missing data issues, we conducted a comprehensive review of electronic medical records and excluded cases with incomplete preoperative nutritional or clinical parameters. Patients with missing follow-up data were censored at the last confirmed contact date. No imputation methods were employed, as the exclusion criteria ensured that only complete datasets were included in the final analysis. This approach aligns with recommendations for retrospective cohort studies, aiming to minimize bias from incomplete information.

### 2.7 Statistical analysis

Statistical analyses were performed using SPSS version 25.0 (IBM Corporation, Armonk, NY, USA) and graphical representations were generated using R version 4.3.2.^1^ All statistical tests were two-sided, and differences with *p* < 0.05 were considered to be significant. The baseline characteristics of the 2 patient cohorts were evaluated using descriptive statistics. Categorical variables were compared using the chi-squared test or Fisher’s exact test, as appropriate. For continuous variables, the Kolmogorov–Smirnov test was first used to assess the normality of data distribution. Normally distributed data are expressed as mean ± standard deviation (SD) and were compared using *t*-tests or analysis of variance. Non-normally distributed data are expressed as median and were compared using rank-sum tests. Survival analyses were performed using the Kaplan–Meier method, with differences in survival rates assessed using the log-rank test. A Cox proportional hazards regression model was used to identify independent prognostic factors influencing OS in patients undergoing hepatectomy. Hazard ratio (HR) with corresponding 95% confidence interval (CI) were calculated for each variable, and nomograms and forest plots were constructed based on independent prognostic factors identified in the training cohort. The predictive performance of the nomogram model was evaluated and validated using the concordance index (C-index), calibration curves, time-dependent area under the curve (AUC), time-dependent receiver operating characteristic (ROC) curves, and decision curve analysis (DCA). Using OS as the primary endpoint, X-tile software was used to determine the optimal cut-off values for ALBI and PNI scores. Patients were subsequently stratified into groups based on these thresholds. Comparative analyses of the nomogram prediction model, BCLC staging system, and ALBI and PNI models were conducted, focusing on ROC curves, AUC values, and DCA metrics.

## 3 Results

### 3.1 Baseline characteristics

This study included 477 patients with hepatocellular carcinoma who underwent hepatectomy, with 297 in the training cohort and 180 in the validation cohort. The training cohort comprised 297 patients, among whom 207 events (deaths) occurred, yielding an EPV ratio of 41.4 for the five independent predictors. In the validation cohort of 180 patients, there were 114 events (deaths). Despite differences in sample size, the two cohorts were comparable in most baseline characteristics. Furthermore, there were a sufficient number of events in the validation cohort to reliably assess the model’s performance. This sample allocation ensures the model’s generalizability while maintaining statistical reliability.

A comparative analysis of baseline characteristics revealed that the training cohort had a significantly higher proportion of patients with preoperative sarcopenia (*p* < 0.05) and a notably shorter postoperative hospital stay (*p* < 0.05) than the validation cohort. No statistically significant differences in other baseline parameters were observed between the 2 cohorts (Table 2). The postoperative hospital stay was significantly shorter in the training cohort compared with the validation cohort [median: 10 days (IQR 8.0–13.0) vs. 11 days (IQR 8.0–14.0), *p* = 0.008]. Although this difference is statistically significant, its clinical relevance may be limited. Notably, there were no significant differences between the two groups in terms of the severity of complications (≤Grade II: 87.2% vs. 91.1%, ≥Grade III: 12.8% vs. 8.9%, *p* = 0.192) or the extent of surgery (<3 liver segments resected: 39.7% vs. 37.2%, ≥3 liver segments resected: 60.3% vs. 62.8%, *p* = 0.586), indicating similar postoperative recovery processes and surgical complexity. Therefore, the one-day difference in median hospital stay more likely reflects variations in postoperative management strategies across different centers rather than substantial differences in patient clinical outcomes.

**Table 2:** Baseline data of patients with HCC in training and validation cohorts.

Characteristics	Total (*n* = 477)	Training cohort (*n* = 297)	Validation cohort (*n* = 180)	Statistics	*p*-value
Gender, *n* (%)				*χ*^2^ = 0.04	0.833
Male	406 (85.1)	252 (84.8)	154 (85.6)		
Female	71 (14.9)	45 (15.2)	26 (14.4)		
Age (years), Mean ± SD^a^	52.50 ± 11.79	51.19 ± 11.03	53.29 ± 12.17	*t* = 1.90	0.059
BMI (kg/m^2^), *n* (%)				–	0.910
Normal	445 (93.3)	278 (93.6)	167 (92.8)		
Moderate reduction	23 (4.8)	14 (4.7)	9 (5.0)		
Severe reduction	9 (1.9)	5 (1.7)	4 (2.2)		
BCLC, *n* (%)				*χ*^2^ = 3.14	0.371
0	38 (8.0)	28 (9.4)	10 (5.6)		
A	318 (66.7)	197 (66.4)	121 (67.2)		
B	59 (12.4)	33 (11.1)	26 (14.4)		
C	62 (12.9)	39 (13.1)	23 (12.8)		
AFP (ng/mL), *n* (%)				*χ*^2^ = 0.01	0.933
<400	293 (61.4)	182 (61.3)	111 (61.7)		
≥400	184 (38.6)	115 (38.7)	69 (38.3)		
Maximum tumor diameter (cm), *n* (%)				*χ*^2^ = 0.07	0.797
>5	258 (54.1)	162 (54.5)	96 (53.3)		
≤5	219 (45.9)	135 (45.5)	84 (46.7)		
Number of tumors, *n* (%)				*χ*^2^ = 4.19	0.123
1	390 (81.8)	251 (84.5)	139 (77.2)		
2	34 (7.1)	17 (5.7)	17 (9.5)		
≥3	53 (11.1)	29 (9.8)	24 (13.3)		
HBsAg, *n* (%)				*χ*^2^ = 1.80	0.18
Negative	86 (18.0)	59 (19.9)	27 (15.0)		
Positive	391 (82.0)	238 (80.1)	153 (85.0)		
Albumin (g/L), *n* (%)				*χ*^2^ = 0.223	0.637
<35	117 (24.5)	75 (25.3)	42 (23.3)		
≥35	360 (75.5)	222 (74.7)	138 (76.7)		
L3-SMI (cm^2^/m^2^), *n* (%)				*χ*^2^ = 6.193	0.013*
Sarcopenia	97 (20.3)	71 (23.9)	26 (14.4)		
Normal	380 (79.7)	226 (76.1)	154 (85.6)		
Unintentional weight loss, *n* (%)				–	0.598
Normal	430 (90.1)	267 (89.9)	163 (90.6)		
Moderate loss	39 (8.2)	26 (8.8)	13 (7.2)		
Severe loss	8 (1.7)	4 (1.3)	4 (2.2)		
GLIM criteria, *n* (%)				*χ*^2^ = 1.42	0.234
Non-malnourished	340 (71.3)	206 (69.4)	134 (74.4)		
Malnourished	137 (28.7)	91 (30.6)	46 (25.6)		
Child-Pugh grade, *n* (%)				*χ*^2^ = 3.82	0.051
A	448 (93.9)	274 (92.3)	174 (96.7)		
B	29 (6.1)	23 (7.7)	6 (3.3)		
Lymphocyte count, M (Q1, Q3)	1.80 (1.39, 2.23)	1.81 (1.47, 2.20)	1.77 (1.37, 2.25)	*Z* = −0.89	0.375
MVI, *n* (%)				*χ*^2^ = 0.01	0.911
No	415 (87.0)	157 (87.2)	258 (86.9)		
Yes	62 (13.0)	23 (12.8)	39 (13.1)		
Complication grade, *n* (%)				*χ*^2^ = 1.703	0.192
≤Grade II	423 (88.7)	259 (87.2)	164 (91.1)		
≥Grade III	54 (11.3)	38 (12.8)	16 (8.9)		
Extent of hepatectomy, *n* (%)				*χ*^2^ = 0.297	0.586
<3 segments	185 (38.8)	118 (39.7)	67 (37.2)		
≥3 segments	292 (61.2)	179 (60.3)	113 (62.8)		
Perioperative allogeneic transfusion, *n* (%)				*χ*^2^ = 0.84	0.359
No	394 (82.6)	249 (83.8)	145 (80.6)		
Yes	83 (17.4)	48 (16.2)	35 (19.4)		
Postoperative hospital stay, M (Q1, Q3)	10.0 (8.0, 14.0)	10.0(8.0, 13.0)	11.00 (8.0, 14.0)	*Z* = −0.068	0.008*

AFP, Alpha-Fetoprotein; BCLC, Barcelona Clinic Liver Cancer; BMI, Body Mass Index; GLIM, Global Leadership Initiative on Malnutrition; HBsAg, Hepatitis B Surface Antigen; HCC, Hepatocellular Carcinoma; L3-SMI, L3 Skeletal Muscle Index; M, Median; MVI, Microvascular Invasion; *n*, number; *p < 0.05; *t*, *t*-test; *Z*, rank sum test; *χ*^2^, chi-square test; −, Fisher’s exact test; SD, standard deviation; M, Median; Q1, 1st Quartile; Q3, 3rd Quartile.

a Normally distributed data.

The primary purpose of establishing a validation cohort was to assess the generalizability and applicability of the model developed using data from the training cohort to an independent non-modeling population. In predictive model development and evaluation, unlike in randomized controlled clinical trials, strictly balanced and comparable baseline characteristics between the training and validation cohorts are not paramount (19).

### 3.2 Survival analysis of the training and validation cohorts

The median follow-up for the training and validation cohorts were 62.0 months (95% CI 57.9–75.4) and 48.6 months (95% CI 45.5–49.6), respectively. As of August 31, 2022, 90 patients in the training cohort were alive, and 207 had died; in the validation cohort, 66 patients survived and 114 died. In the training cohort, the 1-, 2-, and 3-year overall survival (OS) rates were 84.2, 58, and 38.5%, respectively. For the validation cohort, these rates were 91.7, 80.6, and 56.8%, respectively.

### 3.3 Impact of malnutrition on OS in patients undergoing hepatectomy with different BCLC stages of HCC

**Figure 1:**
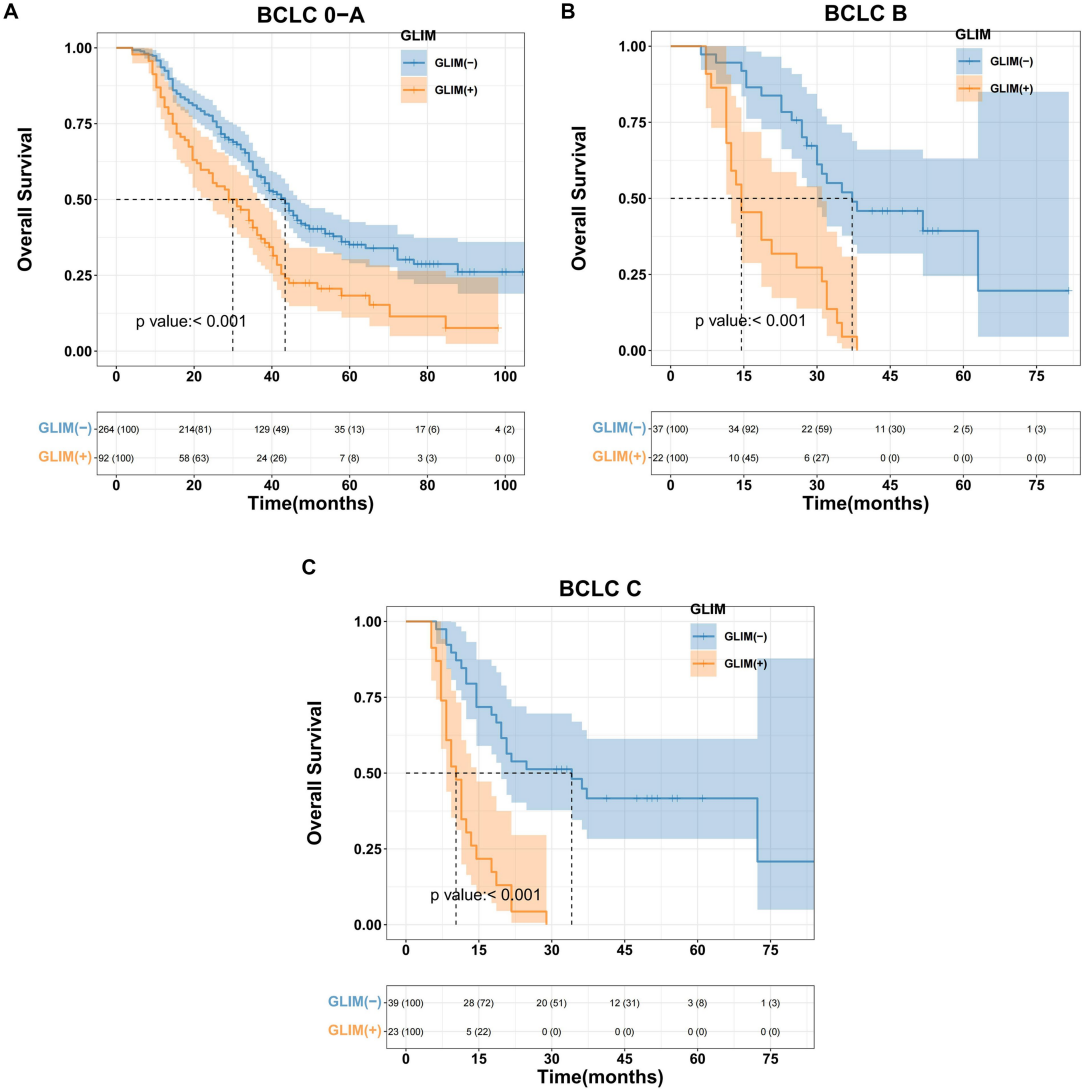
Kaplan–Meier curves for overall survival based on GLIM criteria of different BCLC stage HCC patients. **(A)** Malnourished patients with BCLC stage 0-A had significantly worse overall survival than non-malnourished patients (*p* < 0.001), **(B)** malnourished patients with BCLC stage B had significantly worse overall survival than non-malnourished patients (*p* < 0.001), **(C)** malnourished patients with BCLC stage C had significantly worse overall survival than non-malnourished patients (*p* < 0.001).

Kaplan–Meier analysis was used to evaluate the influence of malnutrition on OS among patients undergoing hepatectomy with varying BCLC stages. The results demonstrated statistically significant differences across all stages. For BCLC stage 0-A, the median survival for the non-malnourished group was 43.4 months (95% CI 38.2–46.5), whereas the malnourished group exhibited a median survival of 29.9 months (95% CI 23.8–36.2); this difference was statistically significant (*p* < 0.001, Figure 1A). For BCLC stage B, the non-malnourished group had a median survival of 37.2 months (95% CI 30.4–41.3), while the median survival in the malnourished group was 14.5 months (95% CI 12.4–31); this difference was statistically significant (*p* < 0.001, Figure 1B). For BCLC stage C, the non-malnourished group exhibited a median survival of 34.1 months (95% CI 19.6–38.5), contrasted with 10.3 months (95% CI 8.3–14.5) for the malnourished group; this difference was also statistically significant (*p* < 0.001, Figure 1C).

### 3.4 Impact of malnutrition on OS in the training cohort

**Figure 2:**
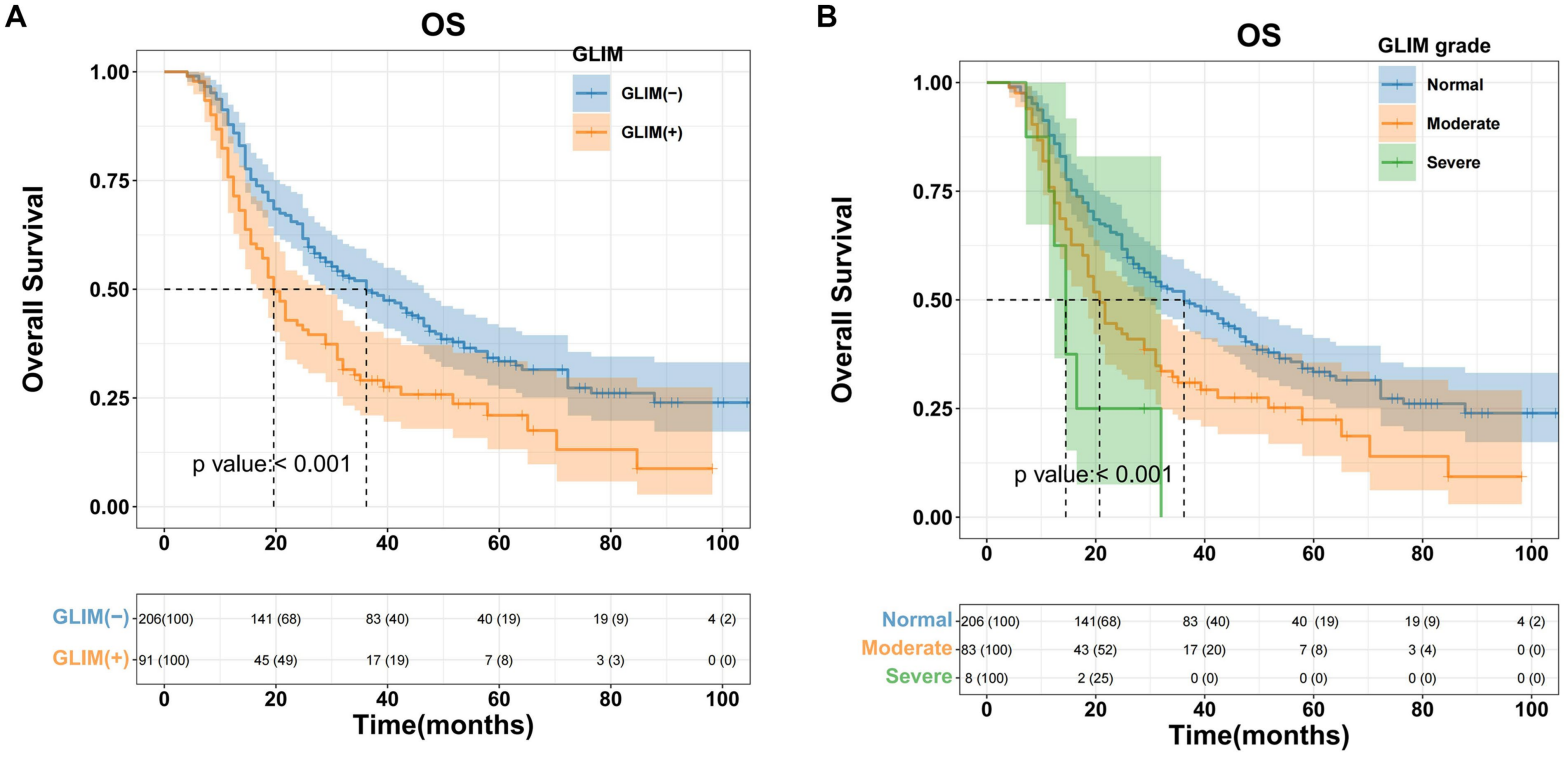
Kaplan–Meier curves for overall survival based on GLIM criteria of HCC patients who underwent hepatectomy in the training cohort. **(A)** Malnourished patients had significantly worse overall survival (OS) than non-malnourished patients (*p* < 0.001), **(B)** patients with moderate/severe malnutrition had significantly worse OS than patients with normal nutrition (*p* < 0.001).

In the training cohort, Kaplan–Meier survival analysis revealed significant disparities in OS between the non-malnourished and malnourished groups. The non-malnourished group exhibited a median OS of 36.2 months (95% CI 28.9–46.5), with 1-, 2-, and 3-year OS rates of 87.9, 65.1, and 44.8%, respectively. In contrast, the malnourished group demonstrated a median OS of 19.6 months (95% CI 17.6–28.9), with corresponding 1-, 2-, and 3-year OS rates of 75.9, 41.9, and 24.4%. The difference in OS between the 2 groups was statistically significant (*p* < 0.001, Figure 2A). Furthermore, a stratified analysis based on the severity of malnutrition yielded additional insights. The moderately malnourished group exhibited a median OS of 20.7 months (95% CI 18.6–31.0), with 1-, 2-, and 3-year OS rates of 75.8, 43.3, and 26.5%, respectively. The severely malnourished group exhibited a markedly reduced median OS of 14.5 months (95% CI 12.4–24.4), with 1-, 2-, and 3-year OS rates of 75, 50, and 0%, respectively. The difference in OS between these 2 subgroups of malnourished patients was also statistically significant (*p* < 0.001, Figure 2B).

### 3.5 Determination of threshold values for inflammatory, nutritional, and immune indicators in the training cohort

Using OS as the primary endpoint, the optimal cut-off values for the ALBI score and PNI were determined using X-tile software (Yale University School of Medicine, New Haven, CT, USA). Based on the established PNI threshold, patients in both the training and validation cohorts were stratified into “high” and “low” groups. Similarly, the ALBI threshold facilitated the categorization of patients in both cohorts into 3 distinct grades (1–3) (Table 3).

**Table 3:** The optimal cut-off values of ALBI and PNI with overall survival as study endpoint.

Variable	Optimal threshold
ALBI	Grade 1: ≤−2.56
	Grade 2: −2.56 <X <−2.28
	Grade 3: ≥−2.28
PNI	46.4

ALBI, Albumin-Bilirubin Index; PNI, Prognostic Nutritional Index.

### 3.6 Univariate and multivariate survival analysis in the training cohort

**Figure 3:**
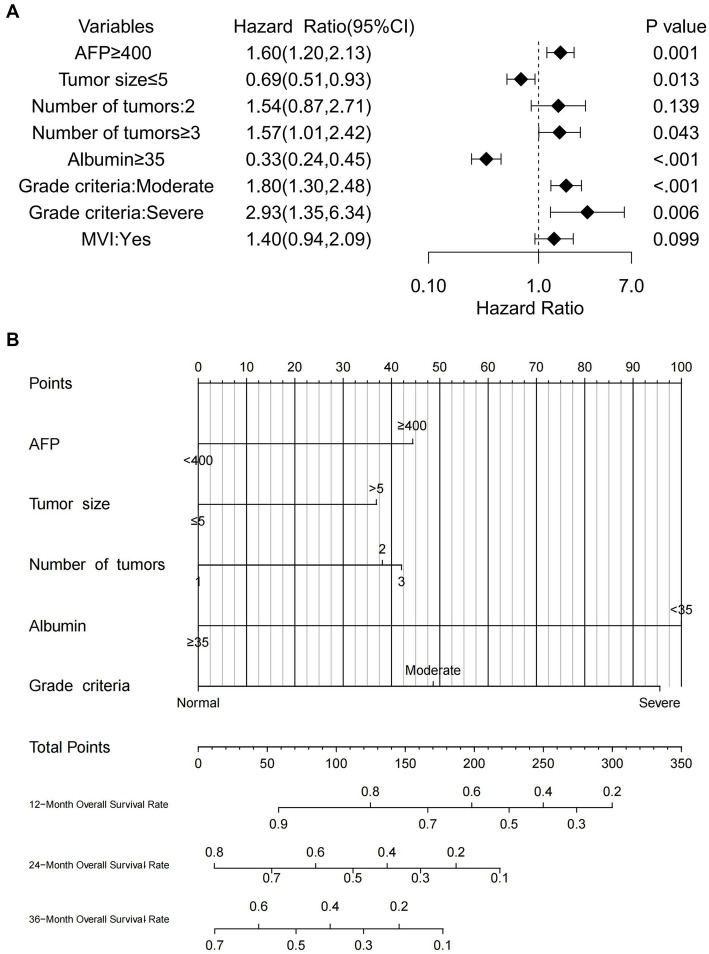
**(A)** Forest plot of OS in HCC patients who underwent hepatectomy in the training cohort, **(B)** nomogram predicting OS in HCC patients who underwent hepatectomy in the training cohort.

Univariate Cox regression analysis revealed several factors significantly associated with poor OS (all *p* < 0.05): AFP ≥ 400 ng/mL; malnutrition (both moderate and severe); serum albumin <35 g/L; maximum tumor diameter >5 cm; tumor number ≥3; and presence of MVI. Subsequent multivariate analysis identified the following independent risk factors influencing OS: AFP ≥ 400 ng/mL (*p* = 0.001); moderate malnutrition (*p* < 0.001); severe malnutrition (*p* = 0.006); serum albumin <35 g/L (*p* < 0.001); maximum tumor diameter >5 cm (*p* = 0.013); and tumor number ≥3 (*p* = 0.043). The findings are summarized in Table 4. Forest plots illustrating the impact of these factors on OS were constructed based on the Cox regression results (Figure 3A).

**Table 4:** Univariate and multivariate COX analysis of overall survival in HCC patients who underwent hepatectomy in training cohort.

Variables	Univariate analysis	*p* value	Multivariate analysis	*p* value
	HR (95% CI)		HR (95% CI)	
AFP (ng/mL)				
<400	Reference		Reference	
≥400	1.738 (1.320, 2.288)	<0.001*	1.601 (1.204, 2.128)	0.001*
GLIM grade criteria				
Non-malnourished	Reference		Reference	
Moderate malnutrition	1.609 (1.193, 2.170)	0.002*	1.797 (1.305, 2.475)	<0.001*
Severe malnutrition	3.068 (1.424, 6.612)	0.004*	2.928 (1.351, 6.343)	0.006*
Chronic hepatitis B				
No	Reference			
Yes	0.959 (0.681, 1.351)	0.812		
Albumin (g/dL)				
<35	Reference		Reference	
≥35	0.324 (0.240, 0.437)	<0.001*	0.328 (0.241, 0.448)	<0.001*
Maximum tumor diameter (cm)				
>5	Reference		Reference	
≤5	0.562 (0.424, 0.743)	<0.001*	0.690 (0.514, 0.926)	0.013*
Number of tumors				
1	Reference		Reference	
2	1.371 (0.780, 2.413)	0.273	1.537 (0.870, 2.714)	0.139
≥3	2.289 (1.506, 3.479)	<0.001*	1.565 (1.013, 2.418)	0.043*
MVI				
No	Reference		Reference	
Yes	2.032 (1.391, 2.968)	<0.001*	1.401 (0.939, 2.091)	0.099
Gender				
Male	Reference			
Female	1.107 (0.755, 1.622)	0.603		

HR, Hazard Ratio; CI, Confidence Interval; AFP, Alpha-Fetoprotein; GLIM, Global Leadership Initiative on Malnutrition; MVI, Microvascular Invasion; *statistically significant (*p* < 0.05).

### 3.7 Development of a nomogram model for predicting post-hepatectomy OS in patients with HCC from the training cohort

Based on the results of multivariate Cox regression analysis, the following predictors were identified for post-hepatectomy OS in patients with HCC: serum albumin, alpha-fetoprotein (AFP), maximum tumor diameter, tumor number, and GLIM malnutrition grade. The nomogram (Figure 3B) enables the calculation of an individual’s total score by summing the points corresponding to each predictor. The total score for OS prediction ranged from 0 to 350. To estimate individual 1-, 2-, and 3-year OS probabilities, a vertical line was drawn from the total points axis to the survival probability axis. This user-friendly design facilitates a rapid and straightforward prognostic assessment for clinicians.

### 3.8 Evaluation of the nomogram model

**Figure 4:**
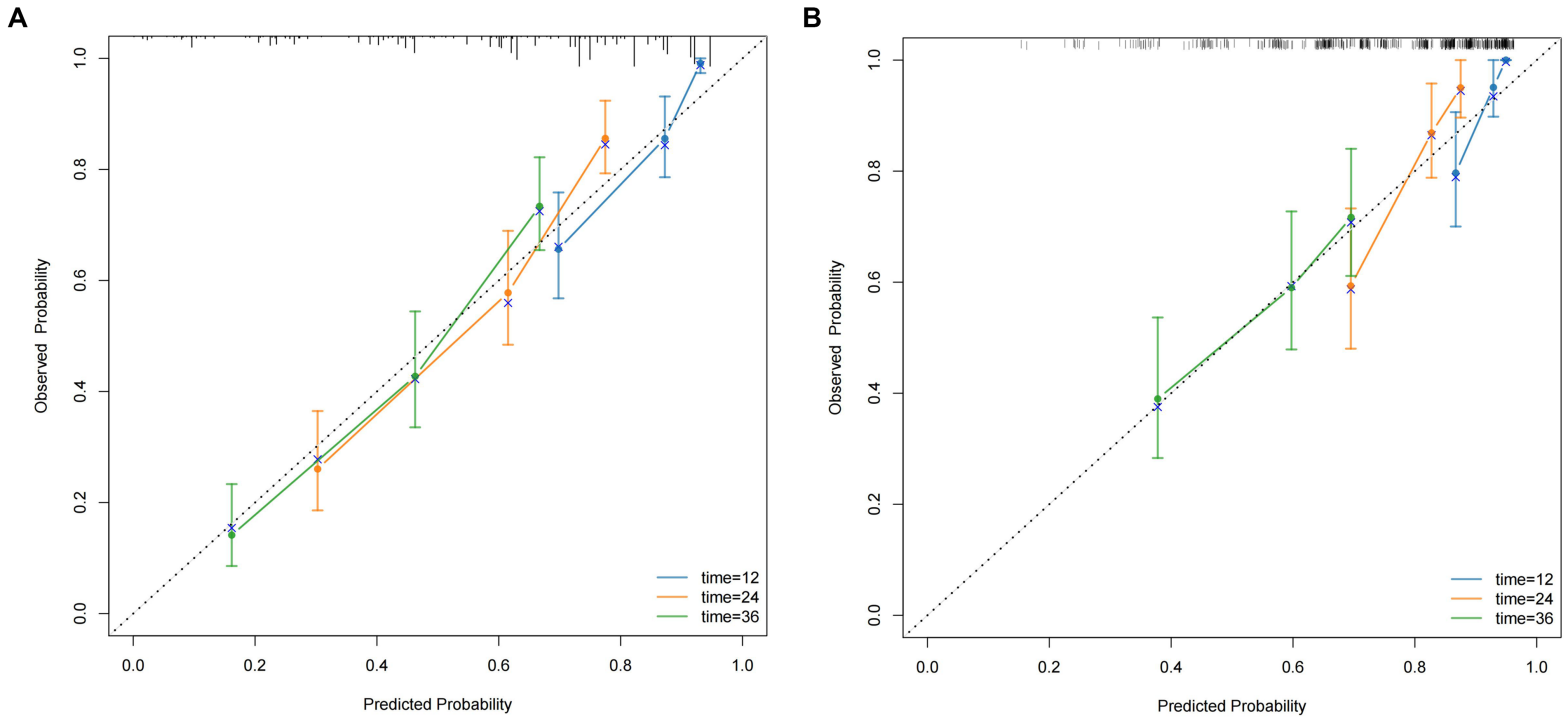
The calibration curves for predicting the 1, 2, and 3-year OS **(A)** of HCC patients who underwent hepatectomy in the training cohort and the 1, 2, and 3-year OS **(B)** of HCC patients who underwent hepatectomy in the validation cohort.

Multiple statistical methods were used to evaluate the performance of the nomogram, including the C-index, calibration curves, time-dependent AUC, time-dependent ROC curves, and DCA. In the training cohort, the C-indices for predicting 1-, 2-, and 3-year OS were 0.793, 0.744, and 0.735, respectively. The validation cohort exhibited C-indices of 0.838, 0.761, and 0.666 at the same time points, and the calibration curves provided a visual representation of the relationship between the predicted and actual probabilities (Figures 4A,B). In the training cohort, these curves demonstrated good concordance between the predicted and observed 1-, 2-, and 3-year OS rates, with no significant deviation from the reference line. However, in the external validation cohort, optimal consistency was observed only for the 1-year OS prediction.

**Figure 5:**
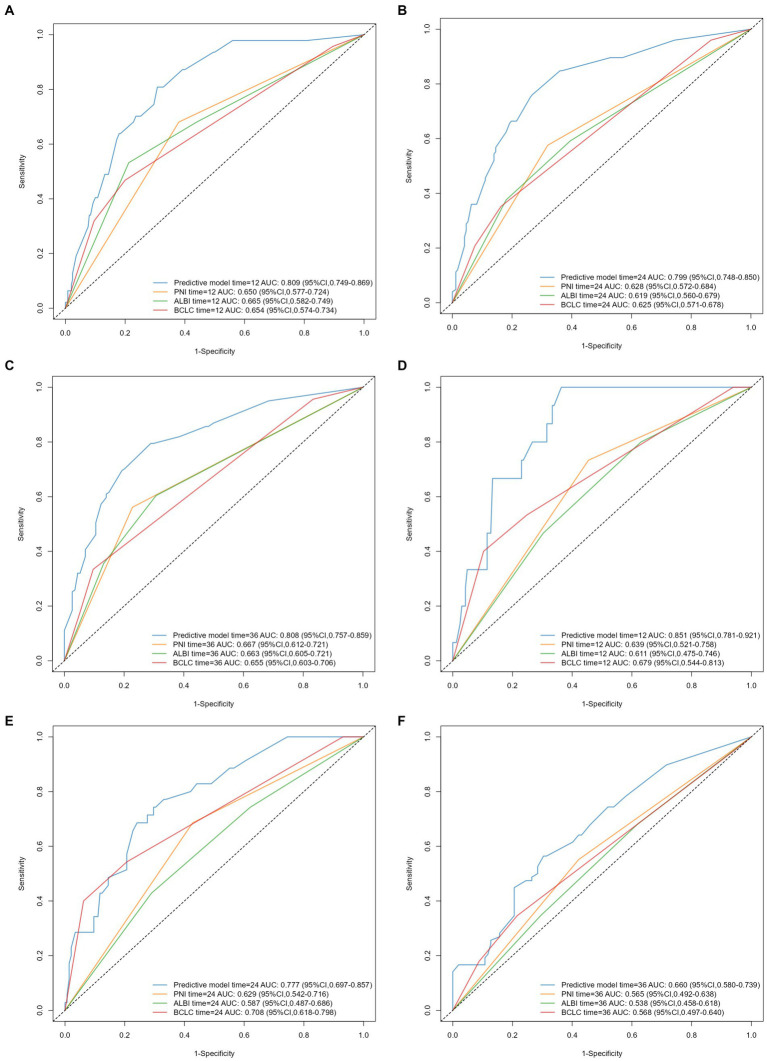
Time-dependent ROC curves and AUC values of predictive models for 1-, 2-, and 3-year OS in HCC patients after hepatectomy: **(A–C)** training cohort, **(D–F)** validation cohort.

**Figure 6:**
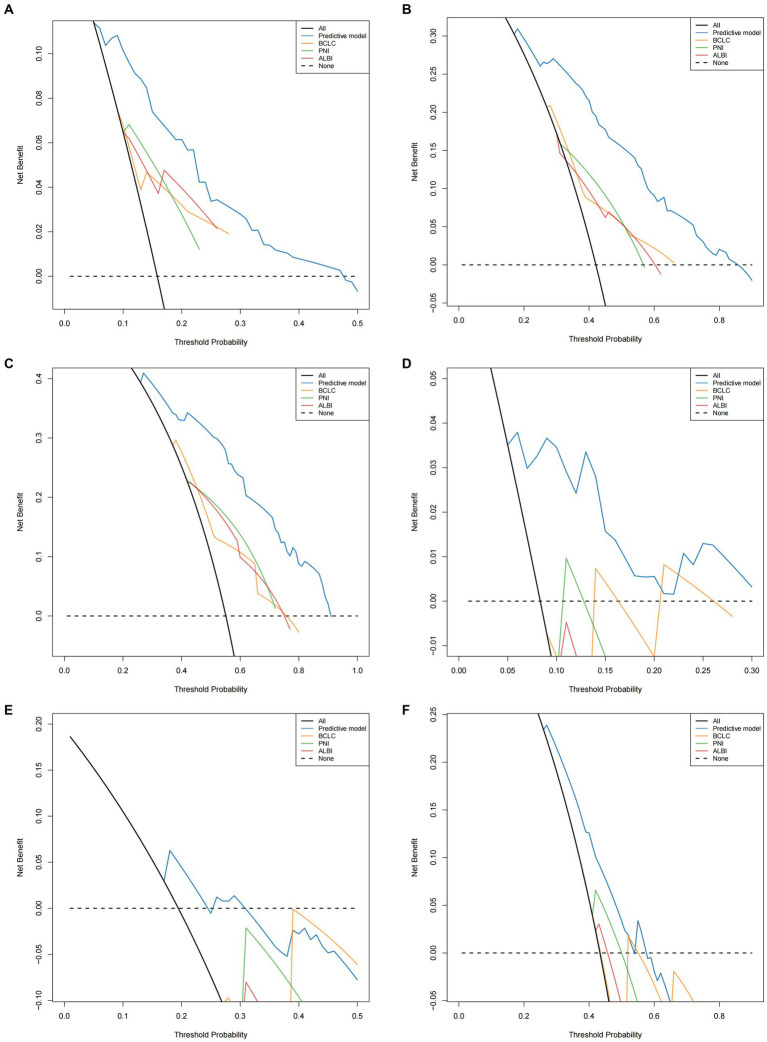
Comparison of the DCA of nomogram model and PNI/ALBI/BCLC stage in predicting OS **(A–C)** of HCC patients who underwent hepatectomy in the training cohort; comparison of the DCA of nomogram model and PNI/ALBI/BCLC stage in predicting OS **(D–F)** of HCC patients who underwent hepatectomy in the validation cohort.

Time-dependent AUC curves in both cohorts revealed the superior accuracy of the nomogram in predicting 1-, 2-, and 3-year OS compared with the ALBI, PNI, and BCLC staging system. Time-dependent ROC curves yielded the following results: training cohort, 1-year OS, AUC 0.809 (95% CI 0.749–0.869, Figure 5A); 2-year OS, AUC 0.799 (95% CI 0.748–0.850, Figure 5B); and 3-year OS, AUC 0.808 (95% CI 0.757–0.859, Figure 5C); validation cohort, 1-year OS, AUC 0.851 (95% CI 0.781–0.921, Figure 5D); 2-year OS, AUC 0.777 (95% CI 0.697–0.857, Figure 5E); 3-year OS, AUC 0.660 (95% CI 0.580–0.739, Figure 5F). Furthermore, the DCA curves demonstrated favorable net benefits when the nomogram was used to predict prognosis in patients with HCC undergoing hepatectomy. In the training cohort, the nomogram exhibited superior predictive ability for 3-year OS across a wider range of reasonable threshold probabilities than the ALBI, PNI, and BCLC staging systems (Figures 6A–C). Similarly, in the validation cohort, the nomogram yielded better net benefits and superior predictive capability for 3-year OS than the ALBI, PNI, and BCLC models (Figures 6D–F).

## 4 Discussion

The nomogram predictive model based on GLIM criteria demonstrated strong discriminative ability, calibration, and clinical applicability. To our knowledge, this study involved the largest sample size to date investigating the impact of malnutrition on post-hepatectomy outcomes in patients with HCC. This is also the first study to develop a preoperative nomogram model based on the GLIM criteria for predicting post-hepatectomy survival outcomes in patients with HCC.

Malnutrition is a common complication in patients with HCC and cirrhosis. Its occurrence is often attributed to metabolic abnormalities caused by tumor factors, where catabolism exceeds anabolism, decreased gastrointestinal function due to portal hypertension leads to reduced intake and absorption of nutrients, and persistent low-grade inflammation causes symptoms such as anorexia and early satiety (20). These factors contribute to the clinical manifestations in patients with HCC, including weight loss, decreased appetite, and reduced muscle mass. As a comprehensive malnutrition diagnostic tool, GLIM encapsulates these critical malnutrition-related clinical presentations. Previous research has revealed good consistency between the GLIM and PG-SGA in diagnosing malnutrition in gastrointestinal tumors (10, 11, 21, 22). Our earlier studies also demonstrated the excellent diagnostic utility of GLIM for malnutrition in patients with HCC. Given the comprehensiveness, objectivity, and retrospective applicability of the GLIM, we chose it to define malnutrition in this study. In our cohort of patients with HCC who underwent hepatectomy, the incidence of malnutrition was 28.7% (137/477 patients). A Japanese study (23) reported a higher incidence (56%) among 293 patients with HCC who underwent hepatectomy. However, that study included a higher proportion of patients with advanced-stage disease [BCLC B/C stage, 35% (104/293)] than our study [12.9% (62/477)]. These results suggest that the incidence of malnutrition increases with tumor progression, emphasizing the need for greater attention to nutritional issues in patients with advanced-stage disease in clinical practice.

In recent years, the influence and predictive value of the GLIM criteria on survival outcomes in malignant tumors have emerged as a focal point in clinical nutrition research. Evidence indicates that malnutrition is an independent risk factor for overall survival in multiple cancers, including gastric, colon, esophageal, and lung cancers (13–15, 24). Additionally, Yin et al. (25) found a significant association between malnutrition and the incidence of postoperative complications in esophageal cancer patients (OR = 7.52, *p* < 0.001). However, the role of GLIM in HCC patients undergoing liver resection remains underexplored. Omiya et al. (23) validated the association between GLIM and HCC prognosis in a single-center study involving 293 patients but did not develop a prognostic model. In contrast, our study applied the GLIM criteria to HCC patients following liver resection and, for the first time, constructed a nomogram model incorporating GLIM. The results revealed that this model outperformed traditional scoring tools in both the training and validation cohorts. Conducted using multi-center data and subjected to rigorous external validation, this study strengthens the evidence supporting the utility and applicability of GLIM in HCC prognosis assessment. In our study, we performed subgroup stratification based on BCLC staging. Results revealed that patients who were malnourished had a significantly poorer OS than those who were non-malnourished across all BCLC stages. Although the BCLC staging system comprehensively incorporates factors, such as tumor burden, liver function, and performance status, it lacks comprehensive nutritional assessment indicators. Our results suggest that, regardless of the BCLC stage, malnutrition is associated with OS, indicating that nutritional status may influence survival outcomes independently of tumor factors. Further multivariate analysis confirmed malnutrition as an independent risk factor for OS in patients with HCC undergoing hepatectomy; however, the specific mechanisms by which nutritional status affects survival prognosis in patients with cancer have not yet been fully elucidated. We posit that under tumor burden and surgical stress, patients with cancer typically experience an inflammatory state, manifesting as cellular homeostasis disruption, organ dysfunction, negative nitrogen balance, and changes in body composition. Faced with these pathological processes, malnourished patients are more susceptible to overall deterioration, potentially developing sarcopenia or cachexia, which can increase the risk for poor prognosis (26, 27). Conversely, good nutritional status may facilitate postoperative adjuvant therapy and rehabilitation, potentially reducing the risk for recurrence and improving survival outcomes. Additionally, nutritional status influences both innate and adaptive immune functions (28), thereby affecting tumor disease progression. We believe that nutritional status, unlike other tumor-related prognostic factors, can be improved, to some extent, through perioperative nutritional management. Therefore, the significance of malnutrition as an independent risk factor for post-hepatectomy prognosis in HCC lies not only in its predictive value for survival, but also in highlighting the importance of perioperative nutritional management and providing a diagnostic basis for nutritional therapy.

Multivariate Cox regression analysis identified the following independent risk factors for OS in patients with HCC undergoing hepatectomy: AFP ≥ 400 ng/mL; Malnutrition; serum albumin <35 g/L; maximum tumor diameter >5 cm; and tumor number ≥3. The impact of maximum tumor diameter on survival outcomes may be attributed to several factors. First, a larger tumor diameter generally necessitates more extensive hepatectomy, increasing surgical difficulty and duration. This may lead to greater blood loss, perioperative blood transfusions, and postoperative complications, affecting patient recovery and potentially reducing tolerance for postoperative adjuvant therapy or treatment upon recurrence. Second, larger tumor diameter increases the risk for MVI. Zhou et al. (29) reported that tumors measuring > 5 cm in diameter significantly increased the risk for MVI in 137 patients with HCC. Another study reported MVI incidence rates of 25, 40, 55, and 63% for HCC sizes <3 cm, 3–5 cm, 5–6.5 cm, and >6.5 cm, respectively (30). The increased incidence of MVI with tumor size may be due to enhanced tumor blood supply demands as the tumor grows, leading to increased microvascular proliferation around the tumor and, consequently, higher probability of MVI. MVI, defined as the presence of metastatic HCC microemboli in intrahepatic blood vessels, is a recognized key determinant of HCC survival. In our study, MVI was a significant factor in the univariate Cox regression analysis for OS, but was not an independent risk factor in the multivariate analysis. This may be due to confounding bias between MVI and tumor size, leading to its exclusion from multivariate analysis. AFP is a well-established and widely used tumor marker for HCC diagnosis and prognosis prediction associated with promoting HCC cell proliferation and vascular invasion (31, 32). Studies have shown that AFP is an independent risk factor for post-hepatectomy survival (33–35), which is consistent with our findings.

In addition to the aforementioned tumor-related indicators, our study identified 2 nutrition-related indicators as independent risk factors for post-hepatectomy OS in patients with HCC: serum albumin level <35 g/L; and Malnutrition. Serum albumin, which accounts for more than one-half of blood proteins, reflects the protein status of the blood and internal organs, and is used clinically as a serum nutritional marker (36). Concurrently, serum albumin is a primary indicator of liver function and is featured in common liver function assessment tools such as the Child–Pugh and ALBI scores. Liver function is widely recognized as a major factor influencing HCC prognosis (37).

Nomograms are visual tools for predicting individual survival outcomes and can be used to predict clinical outcomes and aid clinical decision making. In this study, we constructed a nomogram predictive model based on independent predictors identified using multivariate Cox regression analysis. This model provided a more intuitive prediction of OS in patients with HCC undergoing hepatectomy. We also conducted external validation of the model’s performance and practical utility. In survival analysis, the C-index is a crucial metric for evaluating a model’s discriminatory ability, with its clinically acceptable range varying depending on the disease’s complexity and clinical context. In the validation cohort of this study, the C-index for 3-year overall survival was 0.666, falling within the range commonly reported for HCC prognostic models (0.52–0.77) (38), indicating a discriminatory ability comparable to other models. Although the C-index in the validation cohort was slightly lower than that in the training cohort, this may be attributed to the smaller sample size. Nonetheless, by incorporating often-overlooked nutritional status data using the Global Leadership Initiative on Malnutrition (GLIM) criteria, the model enhanced its prognostic capability, outperforming the BCLC staging, PNI, and ALBI score in time-dependent ROC analysis and decision curve analysis (DCA). This advancement offers valuable insights for identifying patients who may benefit from nutritional interventions, and the C-index level confirms that the model possesses clinically acceptable efficacy. In terms of predictive ability, the OS prediction model demonstrated good overall predictive accuracy, with corresponding calibration curves indicating good consistency between predicted and observed results. Moreover, the OS prediction model exhibited superior predictive performance for OS in time-dependent ROC analysis compared with the internationally recognized BCLC staging system (39), and common inflammatory-nutritional prognostic indicators, such as PNI (40–42) and ALBI (41, 43). This performance advantage was maintained over time. DCA further demonstrated that our OS prediction model yielded better net benefits than the BCLC staging system, PNI, and ALBI, thus supporting its good clinical applicability. We believe that the superior performance of our nomogram predictive model compared with the 3 reference models is due to its inclusion of both tumor-related factors (e.g., AFP level, maximum tumor diameter and tumor number) and nutritional indicators (e.g., albumin level, GLIM criteria), which are not present in other models. Additionally, the comprehensiveness of the GLIM criteria for nutritional diagnosis may have contributed to the enhanced predictive performance of the model. Furthermore, the indicators used to construct this predictive model were all preoperative, making it applicable for preoperative prediction, which is more meaningful than postoperative prediction.

The novelty of this study lies in the construction of a preoperative nomogram model based on the GLIM criteria for predicting prognosis in patients with HCC undergoing hepatectomy, and the validation of its prognostic predictive ability. This provides a new reference for preoperative screening of potential beneficiaries of surgical treatment. In this study, we excluded patients who received preoperative transarterial chemoembolization (TACE) or postoperative targeted immunotherapy to maintain homogeneity in the study population regarding treatment history. Preoperative TACE can influence postoperative prognosis by inducing tumor necrosis and inflammatory responses, thereby altering tumor biology and the patient’s overall health status (44). Similarly, targeted and immunotherapy are typically used in patients with high recurrence risk or advanced disease, whose baseline characteristics and treatment responses may differ significantly from those who did not receive such treatments (45). These interventions introduce potential confounding factors that may obscure the prognostic impact of malnutrition following radical hepatectomy. By excluding these patients, we minimized bias from treatment-related effects, thereby enhancing the reliability and specificity of our findings regarding the role of GLIM-defined malnutrition in postoperative survival outcomes.

However, this study also had some limitations, the first of which was its retrospective design and inevitable selection bias. Second, the GLIM diagnostic phenotypic criteria use L3-SMI to evaluate muscle mass reduction, which remains cumbersome. Further research is needed to determine whether the predictive value of the GLIM criteria would change significantly if simpler anthropometric or body composition analysis methods were used instead of L3-SMI.

## 5 Conclusion

Results of the present study demonstrated that malnutrition, as diagnosed using the GLIM criteria, is significantly associated with survival outcomes in patients with HCC undergoing hepatectomy. The following independent risk factors for post-hepatectomy OS in patients with HCC were identified: AFP ≥ 400 ng/mL; Malnutrition; serum albumin <35 g/L; maximum tumor diameter >5 cm; tumor and number ≥3. Based on these independent risk factors, a nomogram model was constructed to predict OS. This model effectively predicted survival outcomes in patients with HCC who underwent hepatectomy.

## Data Availability

The raw data supporting the conclusions of this article will be made available by the authors, without undue reservation.

## Footnotes

^1^ http://www.r-project.org/

## References

[1] Sung H Ferlay J Siegel RL Laversanne M Soerjomataram I Jemal A. Global Cancer Statistics 2020: globocan estimates of incidence and mortality worldwide for 36 cancers in 185 countries. CA Cancer J Clin. (2021) 71:209–49. doi: 10.3322/caac.21660, PMID: 33538338

[2] Forner A Reig M Bruix J. Hepatocellular carcinoma. Lancet. (2018) 391:1301–14. doi: 10.1016/S0140-6736(18)30010-2, PMID: 29307467

[3] Drefs M Schoenberg MB Börner N Koliogiannis D Koch DT Schirren MJ. Changes of long-term survival of resection and liver transplantation in hepatocellular carcinoma throughout the years: a meta-analysis. Eur J Surg Oncol. (2024) 50:107952. doi: 10.1016/j.ejso.2024.107952, PMID: 38237275

[4] Arends J Bachmann P Baracos V Barthelemy N Bertz H Bozzetti F. Espen guidelines on nutrition in cancer patients. Clin Nutr. (2017) 36:11–48. doi: 10.1016/j.clnu.2016.07.015, PMID: 27637832

[5] Shen J Dai S Li Z Dai W Hong J Huang J. Effect of enteral immunonutrition in patients undergoing surgery for gastrointestinal cancer: an updated systematic review and meta-analysis. Front Nutr. (2022) 9:941975. doi: 10.3389/fnut.2022.941975, PMID: 35845793 PMC9277464

[6] Kondrup J Allison SP Elia M Vellas B Plauth M. Espen guidelines for nutrition screening 2002. Clin Nutr. (2003) 22:415–21. doi: 10.1016/s0261-5614(03)00098-0 12880610

[7] Persson C Sjödén PO Glimelius B. The Swedish version of the patient-generated subjective global assessment of nutritional status: gastrointestinal vs urological cancers. Clin Nutr. (1999) 18:71–7. doi: 10.1016/s0261-5614(99)80054-5, PMID: 10459083

[8] Bokhorst-De Van Der Schueren MA Guaitoli PR Jansma EP De Vet HC. Nutrition screening tools: does one size fit all? A systematic review of screening tools for the hospital setting. Clin Nutr (2014) 33:39–58. doi: 10.1016/j.clnu.2013.04.008, PMID: 23688831

[9] Jensen GL Cederholm T Correia M Gonzalez MC Fukushima R Higashiguchi T. Glim criteria for the diagnosis of malnutrition: a consensus report from the global clinical nutrition community. JPEN J Parenter Enteral Nutr. (2019) 43:32–40. doi: 10.1002/jpen.1440, PMID: 30175461

[10] Tan S Jiang J Qiu L Liang Y Meng J Tan N. Prevalence of malnutrition in patients with hepatocellular carcinoma: a comparative study of GLIM criteria, NRS2002, and PG-SGA, and identification of independent risk factors. Nutr Cancer. (2024) 76:335–44. doi: 10.1080/01635581.2024.2314317, PMID: 38379140

[11] Zhou L Fu J Ding Z Jin K Wu R Ye LX. Comparison of GLIM, SGA, PG-SGA, and PNI in diagnosing malnutrition among hepatobiliary-pancreatic surgery patients. Front Nutr. (2023) 10:1116243. doi: 10.3389/fnut.2023.1116243, PMID: 36761215 PMC9902504

[12] Tan S Wang J Zhou F Tang M Xu J Zhang Y. Validation of Glim malnutrition criteria in cancer patients undergoing major abdominal surgery: a large-scale prospective study. Clin Nutr. (2022) 41:599–609. doi: 10.1016/j.clnu.2022.01.010, PMID: 35124467

[13] Huang DD Wu GF Luo X Song HN Wang WB Liu NX. Value of muscle quality, strength and gait speed in supporting the predictive power of GLIM-defined malnutrition for postoperative outcomes in overweight patients with gastric cancer. Clin Nutr. (2021) 40:4201–8. doi: 10.1016/j.clnu.2021.01.038, PMID: 33583658

[14] Song HN Wang WB Luo X Huang DD Ruan XJ Xing CG. Effect of GLIM-defined malnutrition on postoperative clinical outcomes in patients with colorectal cancer. Jpn J Clin Oncol. (2022) 52:466–74. doi: 10.1093/jjco/hyab215, PMID: 35062024

[15] Wang P Chen X Liu Q Liu X Li Y. Good performance of the global leadership initiative on malnutrition criteria for diagnosing and classifying malnutrition in people with esophageal cancer undergoing esophagectomy. Nutrition. (2021) 91-92:111420. doi: 10.1016/j.nut.2021.111420, PMID: 34399403

[16] Peduzzi P Concato J Feinstein AR Holford TR. Importance of events per independent variable in proportional hazards regression analysis. II. Accuracy and precision of regression estimates. J Clin Epidemiol. (1995) 48:1503–10. doi: 10.1016/0895-4356(95)00048-8, PMID: 8543964

[17] Collins GS Ogundimu EO Altman DG. Sample size considerations for the external validation of a multivariable prognostic model: a resampling study. Stat Med. (2016) 35:214–26. doi: 10.1002/sim.6787, PMID: 26553135 PMC4738418

[18] Nishikawa H Shiraki M Hiramatsu A Moriya K Hino K Nishiguchi S. Japan Society of Hepatology guidelines for sarcopenia in liver disease (1st edition): recommendation from the working group for creation of sarcopenia assessment criteria. Hepatol Res. (2016) 46:951–63. doi: 10.1111/hepr.12774, PMID: 27481650

[19] Wolff RF Moons KGM Riley RD Whiting PF Westwood M Collins GS. Probast: a tool to assess the risk of bias and applicability of prediction model studies. Ann Intern Med. (2019) 170:51–8. doi: 10.7326/m18-1376 30596875

[20] Laviano A Koverech A Mari A. Cachexia: clinical features when inflammation drives malnutrition. Proc Nutr Soc. (2015) 74:348–54. doi: 10.1017/s0029665115000117, PMID: 25809872

[21] da Silva Couto A Gonzalez MC Martucci RB Feijó PM Rodrigues VD de Pinho NB. Predictive validity of Glim malnutrition diagnosis in patients with colorectal cancer. JPEN J Parenter Enteral Nutr. (2023) 47:420–8. doi: 10.1002/jpen.2475, PMID: 36645343

[22] Qin L Tian Q Zhu W Wu B. The validity of the GLIM criteria for malnutrition in hospitalized patients with gastric cancer. Nutr Cancer. (2021) 73:2732–9. doi: 10.1080/01635581.2020.1856894, PMID: 33305620

[23] Omiya S Urade T Komatsu S Kido M Kuramitsu K Yanagimoto H. Impact of GLIM criteria-based malnutrition diagnosis on outcomes following liver resection for hepatocellular carcinoma. HPB (Oxford). (2023) 25:1555–65. doi: 10.1016/j.hpb.2023.08.012, PMID: 37684130

[24] Yin L Lin X Li N Zhang M He X Liu J. Evaluation of the global leadership initiative on malnutrition criteria using different muscle mass indices for diagnosing malnutrition and predicting survival in lung cancer patients. JPEN J Parenter Enteral Nutr. (2021) 45:607–17. doi: 10.1002/jpen.1873, PMID: 32386328

[25] Yin L Cheng N Chen P Zhang M Li N Lin X. Association of malnutrition, as defined by the PG-SGA, ESPEN 2015, and GLIM criteria, with complications in esophageal cancer patients after esophagectomy. Front Nutr. (2021) 8:632546. doi: 10.3389/fnut.2021.632546, PMID: 33981719 PMC8107390

[26] Rich NE Phen S Desai N Mittal S Yopp AC Yang JD. Cachexia is prevalent in patients with hepatocellular carcinoma and associated with worse prognosis. Clin Gastroenterol Hepatol. (2022) 20:e1157–69. doi: 10.1016/j.cgh.2021.09.022, PMID: 34555519 PMC8934317

[27] Harimoto N Shirabe K Yamashita YI Ikegami T Yoshizumi T Soejima Y. Sarcopenia as a predictor of prognosis in patients following hepatectomy for hepatocellular carcinoma. Br J Surg. (2013) 100:1523–30. doi: 10.1002/bjs.9258, PMID: 24037576

[28] Alwarawrah Y Kiernan K MacIver NJ. Changes in nutritional status impact immune cell metabolism and function. Front Immunol. (2018) 9:1055. doi: 10.3389/fimmu.2018.01055 29868016 PMC5968375

[29] Zhou J Zhang Z Zhou H Leng C Hou B Zhou C. Preoperative circulating tumor cells to predict microvascular invasion and dynamical detection indicate the prognosis of hepatocellular carcinoma. BMC Cancer. (2020) 20:1047. doi: 10.1186/s12885-020-07488-8, PMID: 33129301 PMC7603758

[30] Deng G Yao L Zeng F Xiao L Wang Z. Nomogram for preoperative prediction of microvascular invasion risk in hepatocellular carcinoma. Cancer Manag Res. (2019) 11:9037–45. doi: 10.2147/cmar.S216178, PMID: 31695495 PMC6816236

[31] Chen T Dai X Dai J Ding C Zhang Z Lin Z. AFP promotes HCC progression by suppressing the HUR-mediated FAS/FADD apoptotic pathway. Cell Death Dis. (2020) 11:822. doi: 10.1038/s41419-020-03030-7, PMID: 33009373 PMC7532541

[32] Notarpaolo A Layese R Magistri P Gambato M Colledan M Magini G. Validation of the AFP model as a predictor of HCC recurrence in patients with viral hepatitis-related cirrhosis who had received a liver transplant for HCC. J Hepatol. (2017) 66:552–9. doi: 10.1016/j.jhep.2016.10.038, PMID: 27899297

[33] Xia Y Yan ZL Xi T Wang K Li J Shi LH. A case-control study of correlation between preoperative serum AFP and recurrence of hepatocellular carcinoma after curative hepatectomy. Hepatogastroenterology. (2012) 59:2248–54. doi: 10.5754/hge11978, PMID: 22366528

[34] Zhang B Zhang B Zhang Z Huang Z Chen Y Chen M. 42,573 cases of hepatectomy in China: a multicenter retrospective investigation. Sci China Life Sci. (2018) 61:660–70. doi: 10.1007/s11427-017-9259-9, PMID: 29417360

[35] Zeng J Zeng J Liu J Zeng J. Development of pre and post-operative nomograms to predict individual survival for ideal liver resection candidates with hepatocellular carcinoma. Liver Int. (2021) 41:2974–85. doi: 10.1111/liv.15042, PMID: 34416088

[36] Loftus TJ Brown MP Slish JH Rosenthal MD. Serum levels of prealbumin and albumin for preoperative risk stratification. Nutr Clin Pract. (2019) 34:340–8. doi: 10.1002/ncp.10271, PMID: 30908744

[37] Johnson PJ Berhane S Kagebayashi C Satomura S Teng M Reeves HL. Assessment of liver function in patients with hepatocellular carcinoma: a new evidence-based approach-the ALBI grade. J Clin Oncol. (2015) 33:550–8. doi: 10.1200/jco.2014.57.9151, PMID: 25512453 PMC4322258

[38] Beumer BR Buettner S Galjart B van Vugt JLA de Man RA IJ JNM. Systematic review and meta-analysis of validated prognostic models for resected hepatocellular carcinoma patients. Eur J Surg Oncol. (2022) 48:492–9. doi: 10.1016/j.ejso.2021.09.012 34602315

[39] Reig M Forner A Rimola J Ferrer-Fàbrega J Burrel M Garcia-Criado Á. BCLC strategy for prognosis prediction and treatment recommendation: the 2022 update. J Hepatol. (2022) 76:681–93. doi: 10.1016/j.jhep.2021.11.018, PMID: 34801630 PMC8866082

[40] Feng H Xu F Zhao Y Jin T Liu J Li R. Prognostic value of combined inflammatory and nutritional biomarkers in HCC within the Milan criteria after hepatectomy. Front Oncol. (2022) 12:947302. doi: 10.3389/fonc.2022.947302, PMID: 36132141 PMC9483162

[41] Liang X Liangliang X Peng W Tao Y Jinfu Z Ming Z. Combined prognostic nutritional index and albumin-bilirubin grade to predict the postoperative prognosis of HBV-associated hepatocellular carcinoma patients. Sci Rep. (2021) 11:14624. doi: 10.1038/s41598-021-94035-5, PMID: 34272447 PMC8285529

[42] Wang D Hu X Xiao L Long G Yao L Wang Z. Prognostic nutritional index and systemic immune-inflammation index predict the prognosis of patients with HCC. J Gastrointest Surg. (2021) 25:421–7. doi: 10.1007/s11605-019-04492-7, PMID: 32026332 PMC7904713

[43] Ruzzenente A De Angelis M Conci S Campagnaro T Isa G Bagante F. The albumin-bilirubin score stratifies the outcomes of child-Pugh class A patients after resection of hepatocellular carcinoma. Transl Cancer Res. (2019) 8:S233–44. doi: 10.21037/tcr.2018.12.10, PMID: 35117104 PMC8798373

[44] Hu R Xu J Wang H Wang J Lei K Zhao X. Impact of preoperative transcatheter arterial chemoembolization (TACE) on postoperative Long-term survival in patients with nonsmall hepatocellular carcinoma: a propensity score matching analysis. BMC Cancer. (2024) 24:190. doi: 10.1186/s12885-024-11978-4, PMID: 38336712 PMC10858462

[45] Greten TF Lai CW Li G Staveley-O'Carroll KF. Targeted and immune-based therapies for hepatocellular carcinoma. Gastroenterology. (2019) 156:510–24. doi: 10.1053/j.gastro.2018.09.051, PMID: 30287171 PMC6340758

